# Association between pre-transplant CONUT score and pneumonia risk after allogeneic stem cell transplantation in acute leukemia

**DOI:** 10.1186/s12890-025-04066-1

**Published:** 2025-12-17

**Authors:** Derya Yenibertiz, Esma Sevil Akkurt, Özlem Düvenci̇ Bi̇rben, Tahir Darçin, Ümit Türk, Mehmet Sinan Dal

**Affiliations:** 1 Department of Pulmonology, University of Health Sciences, Dr. Abdurrahman Yurtaslan Ankara Oncology Training and Research Hospital, Ankara, 06200 Türkiye; 2 Department of Hematology, University of Health Sciences, Dr. Abdurrahman Yurtaslan Ankara Oncology Training and Research Hospital, Ankara, 06200 Türkiye

**Keywords:** Allogeneic stem cell transplantation, CONUT score, Pneumonia, Acute leukemia

## Abstract

**Background:**

The Controlling Nutritional Status (CONUT) score has been proposed as a simple tool for assessing nutritional and immunological status and has been associated with prognosis in various malignancies. This study aimed to assess the relationship between pre-transplant CONUT scores and pneumonia development within one year after allogeneic stem cell transplantation (allo-SCT) in patients with acute leukemia.

**Methods:**

In this retrospective single-center study, 158 patients who underwent allo-SCT for acute leukemia between 2013 and 2023 were included. Patients who developed pneumonia during their 1-year post-transplant follow-up were evaluated and their nutritional status was calculated using the CONUT score. The CONUT score was calculated from serum albumin, total cholesterol, and total lymphocyte count measured prior to transplantation. Pneumonia diagnosis was based on thoracic CT findings consistent with clinical symptoms (new infiltrate or consolidation), with microbiological confirmation when available.

**Results:**

Pneumonia occurred in 40 patients (25.3%) within one year after transplantation. The one-year mortality rate was 42.5% (*n* = 17) in patients with pneumonia compared to 22.9% (*n* = 27) in those without pneumonia. Although mortality was higher among patients with pneumonia, no statistically significant association was found between pre-transplant CONUT scores and pneumonia development.

**Conclusion:**

To our knowledge, this is the first study to examine the association between pre-transplant CONUT scores and pneumonia risk after allo-SCT in acute leukemia patients. Our findings indicate no significant predictive value. The multifactorial etiology of pneumonia and the dynamic nutritional and immunological changes in this high-risk population may limit the usefulness of static nutritional indices such as the CONUT score in predicting infectious complications. Incorporating supplemental nutritional or immunological markers may improve risk assessment in this population.

##  Introduction

Allogeneic stem cell transplantation (allo-SCT) is a potentially curative treatment option for patients with acute leukemia; however, post-transplant complications, particularly infections, remain a significant cause of morbidity and mortality [1]. Among these, pneumonia represents one of the most frequent and life-threatening complications, leading to prolonged hospitalization and increased mortality [2]. In recent years, the role of nutritional status in influencing infection risk and clinical outcomes has gained increasing recognition [3].

Cancer patients are particularly vulnerable to malnutrition, as both the disease itself and its treatment compromise their nutritional status. Malnutrition has been associated with adverse outcomes in cancer patients, especially those undergoing allo-SCT. The Controlling Nutritional Status (CONUT) score has been proposed as a simple and effective tool for assessing patients’ nutritional status [4]. The CONUT score, which is based on serum albumin, total cholesterol, and absolute lymphocyte count, has been strongly associated with prognosis in cancer patients. Numerous studies have highlighted the significance of the CONUT score in hematological malignancies [5, 6].

The aim of this study was to evaluate the association between the CONUT score and the development of post-transplant pneumonia among acute leukemia patients undergoing allo-SCT. Clarifying this association could contribute to identifying high-risk patients and guiding early nutritional or preventive interventions to improve post-transplant outcomes.

## Materials and methods

Study population 

This study included 158 patients with a diagnosis of acute leukemia who underwent allogeneic stem cell transplantation (allo-SCT) between 2013 and 2023. Patients aged 18 years or older with a confirmed diagnosis of acute leukemia who underwent allo-SCT were included. Exclusion criteria included age < 18 years, the presence of multiple malignancies, or insufficient medical records preventing access to relevant data.

Data collection

Clinical data, comorbidities, laboratory parameters, chest radiography and thoracic computed tomography findings, pathological results, treatment details, clinical course, and mortality outcomes were retrospectively collected from medical records.

Definition of pneumonia

The diagnosis of pneumonia was based on thoracic computed tomography (CT) findings compatible with clinical symptoms (new infiltrate or consolidation); microbiological confirmation was added when available. Data on immunosuppressive therapy (such as tacrolimus, cyclosporine, or corticosteroids) and graft-versus-host disease (GVHD) status were not available for all patients and therefore were not included in the analysis. Information on conditioning regimen intensity and duration of neutropenia was not systematically documented in patient records.

Nutritional assessment

The Controlling Nutritional Status (CONUT) score is a nutritional grading method based on laboratory test results, including serum albumin (Alb), lymphocyte count (T-Lymph), and total cholesterol (T-Cho) [7]. Scores for reductions in lymphocyte count and cholesterol ranged from 0 to 3, while reductions in albumin were scored from 0 to 6 according to severity. The overall score was categorized into four levels: normal (0–1), mild malnutrition (2–4), moderate malnutrition (5–8), and severe malnutrition (> 9). Higher scores indicated poorer nutritional status. 

Ethical approval

The study was approved by the institutional Ethics Committee (No: 2024-05/65) and conducted in accordance with the Declaration of Helsinki (revised 2013). No organs or tissues were harvested from prisoners for this study. All allogeneic hematopoietic stem cells were obtained from voluntary and eligible donors through the University of Health Sciences, Dr. Abdurrahman Yurtaslan Ankara Oncology Training and Research Hospital, Department of Hematology and Bone Marrow Transplantation Unit, which is an accredited center for donor evaluation, screening, and stem cell collection. All donor procedures were conducted in accordance with national regulations and institutional ethical standards.

### Statistical analysis

Data analysis was performed using IBM SPSS Statistics version 15.0 (SPSS Inc., Chicago, IL, USA). Descriptive statistics were presented as mean ± standard deviation for normally distributed variables, median (min–max) for non-normally distributed variables, and counts and percentages for categorical variables. For comparisons between two groups, the Student’s *t*-test was used for normally distributed variables and the Mann–Whitney *U* test for non-normally distributed variables. For comparisons among more than two groups, ANOVA was used for normally distributed variables and the Kruskal–Wallis test for non-normally distributed variables. Categorical variables were evaluated using Pearson’s Chi-square or Fisher’s exact test, as appropriate. Correlations between continuous variables were assessed with Pearson’s or Spearman’s correlation tests, depending on data distribution. Multivariate analysis was not performed due to the limited number of pneumonia events, which might restrict statistical power. Statistical significance was set at *p* < 0.05.

## Results

Our study included 158 patients who underwent allogeneic stem cell transplantation (allo-SCT) for acute leukemia between 2013 and 2023. Pneumonia developed in 40 patients (25.3%) within one year after transplantation. Among those with pneumonia, 70.0% were male and 30.0% were female. The median age of patients with pneumonia was 42.0 years, compared to 31.5 years in those without pneumonia, and this difference was statistically significant (*p* = 0.041). Similarly, the median age at transplantation was 42.0 years in patients with pneumonia and 27.5 years in those without pneumonia, also reaching statistical significance (*p* = 0.042). By contrast, the median BMI did not differ significantly between the pneumonia and non-pneumonia groups (25.10 vs. 24.36; *p* = 0.383) (Table 1).

**Table 1:** Comparison of demographic characteristics between patients with and without pneumonia within one year after allogeneic stem cell transplantation

Variable	Patients with Pneumonia (*n* = 40)	Patients without Pneumonia (*n* = 118)	*p*-value
Gender (Male/Female)	70.0%/30.0%	60.2%/39.8%	0.267
Median Age (years)	42.00	31.50	0.041*
Median Age at Transplantation (years)	42.00	27.50	0.042*
Median BMI (kg/m²)	25.10	24.36	0.383

*Abbreviations:* BMI: Body mass index

*: *p *< 0.05 was considered statistically significant

The one-year mortality rate was higher among patients with pneumonia (42.5%, n=17) than among those without pneumonia (22.9%, n=27). Bacterial pneumonia, in contrast to other types (fungal, viral, or mixed), was associated with relatively lower mortality rates.

When patients were stratified by CONUT score categories, 5 individuals (4.2%) without pneumonia were in the normal group, while no pneumonia cases were observed in this category. In the mild malnutrition group, 43 patients (36.4%) did not develop pneumonia compared to 18 patients (45.0%) who did. In the moderate malnutrition group, 68 patients (57.6%) did not develop pneumonia, while 20 patients (50.0%) did. The severe malnutrition group included 2 patients (1.7%) without pneumonia and 2 patients (5.0%) with pneumonia. Overall, no statistically significant association was found between CONUT score categories and pneumonia development (χ² = 3.863; *p* = 0.277) (Table 2).

**Table 2:** Distribution of pneumonia development according to CONUT score categories

CONUT Score Category	No Pneumonia (*n* = 118)	Pneumonia (*n* = 40)	Total (*n* = 158)
Normal (0–1)	5 (4.2%)	0 (0.0%)	5 (3.2%)
Mild malnutrition (2–4)	43 (36.4%)	18 (45.0%)	61 (38.6%)
Moderate malnutrition (5–8)	68 (57.6%)	20 (50.0%)	88 (55.7%)
Severe malnutrition (9–12)	2 (1.7%)	2 (5.0%)	4 (2.5%)
Total	118 (100%)	40 (100%)	158 (100%)

*Abbreviations: CONUT:* Controlling Nutritional Status

Statistical test: χ² = 3.863; *p* = 0.277

Note: *p *< 0.05 was considered statistically significant

## Discussion

 Acute leukemia is a serious hematologic disease treated with chemotherapy and allo-SCT. However, these treatments can lead to complications such as serious infections, especially pneumonia. Pneumonia remains a major cause of morbidity and mortality among post-transplant infections, with studies reporting that infections account for up to 40% of non-relapse mortality in allo-SCT patients [7]. Both inflammation and nutritional status play crucial roles in the development of complications such as pneumonia, as malnutrition can impair immune function and increase susceptibility to infections [8].

 The CONUT score, which combines nutritional and immunological indicators, is often used to assess the overall health status of patients. It is calculated based on serum albumin, total lymphocyte count, and total cholesterol levels, providing a simple and cost-effective method to evaluate nutritional and immunological status [4]. Previous studies have shown that the CONUT score correlates with poor outcomes in various malignancies, including hematologic cancers, and can predict overall survival and treatment-related complications [9, 10].

 However, its ability to predict specific infectious complications, particularly pneumonia after allo-SCT for acute leukemia, remains uncertain. In our study, no statistically significant association was found between CONUT score categories and pneumonia development. The occurrence of pneumonia after allo-SCT is multifactorial, influenced by factors such as graft-versus-host disease (GVHD), duration of immunosuppressive therapy, conditioning regimen intensity, and patient age, all of which may attenuate the observed effect size between CONUT score and infection risk [11].

 A meta-analysis investigating the prognostic effect of the CONUT score in patients with hematological malignancies included six studies with 1,811 patients from Japan, Turkey, and China. The median age of patients ranged from 56 to 76 years, and a high CONUT score was identified as a risk factor. The analysis concluded that patients with hematological malignancies and poor nutritional status had an increased mortality risk [9, 12–16]. In our study of 158 acute leukemia patients undergoing allo-SCT, 40 developed pneumonia within the first year after transplantation. Patients with pneumonia had a higher median age than those without pneumonia (42 vs. 31.5 years), and this difference was statistically significant.

 A study by Ureshino et al. investigated the relationship between the CONUT score and survival outcomes in adult T-cell leukemia/lymphoma (ATL) patients undergoing transplantation. They found that the one-year overall survival (OS) and non-relapse mortality (NRM) were significantly associated with the CONUT score [17]. In our study, although one-year mortality was higher in patients with pneumonia, there was no statistically significant difference in pre-transplant CONUT scores between patients with and without pneumonia. Our findings indicate that the CONUT score is not a reliable predictor of pneumonia development in acute leukemia patients after allo-SCT. This result is consistent with some previous studies that have questioned the utility of the CONUT score in predicting infectious complications [18]. While the CONUT score remains useful for assessing nutritional and inflammatory status, its predictive value for pneumonia risk in this clinical setting appears limited rather than completely absent. In patients with acute leukemia undergoing transplantation, nutritional and immune parameters are often already impaired due to the underlying disease, intensive chemotherapy, malnutrition, infections, and immunosuppression. These factors may reduce the accuracy of the CONUT score in reflecting the true clinical condition. The Prognostic Nutritional Index (PNI), which integrates serum albumin and lymphocyte count, may serve as an additional or alternative marker to enhance infection risk prediction in allo-SCT recipients [19]. In summary, this is the first study to specifically evaluate the relationship between the CONUT score and pneumonia development within one year of allo-SCT in acute leukemia patients. Despite its recognized prognostic role in various malignancies, our findings did not demonstrate a significant association between pre-transplant CONUT scores and pneumonia risk. The multifactorial etiology of pneumonia—including nutritional status, immune response, microbial virulence, and the effectiveness of antimicrobial therapies—may limit the value of static nutritional indices such as the CONUT score in this setting [20]. Additionally, specific characteristics of our patient population may have influenced the results.

 Several limitations should be acknowledged. First, the relatively small sample size and single-center design may restrict the generalizability of our findings. Second, the retrospective nature of the study is subject to inherent limitations, including missing data and variability in clinical management. Moreover, the relatively small number of pneumonia events may have limited the statistical power of our analysis, thereby reducing the ability to detect small effect sizes. Although no significant association was found between CONUT scores and pneumonia development, nutritional status remains a critical determinant of post-transplant outcomes. Clinically, our results emphasize the importance of comprehensive nutritional and immunological evaluation before transplantation to identify high-risk patients and to implement targeted nutritional or preventive interventions. Our findings highlight the limited predictive role of the CONUT score for infectious complications in this population and contribute to the existing literature by clarifying its constraints in allo-SCT recipients with acute leukemia. Future large-scale, multicenter prospective studies are warranted to validate these results and to explore additional risk factors, including immunological markers and microbiological profiles, that may better predict post-transplant pneumonia.

## Conclusion

 To our knowledge, this is the first study to evaluate the association between the CONUT score and pneumonia development within the first year after allo-SCT in patients with acute leukemia. Our findings revealed no significant relationship between pre-transplant CONUT scores and subsequent pneumonia occurrence. The variability of score components in this clinical setting, together with the complex and multifactorial nature of pneumonia pathogenesis in allo-SCT recipients, may explain these results. Consequently, the CONUT score alone may have limited predictive value for infectious complications in this patient group. Future research should focus on integrating additional immunological and clinical parameters to improve risk stratification and optimize patient management after transplantation. 

## Data Availability

The data generated and analyzed during this study are available from the corresponding author upon reasonable request.

## Declarations

### Use of Generative AI and AI-assisted Technologies

During the preparation of this manuscript, the authors used ChatGPT to improve the clarity and accuracy of the English language. The authors reviewed and edited the content as necessary and take full responsibility for the content of the publication.
